# Shifting from “Community-Placed” to “Community-Based” Research to Advance Health Equity: A Case Study of the Heatwaves, Housing, and Health: Increasing Climate Resiliency in Detroit (HHH) Partnership

**DOI:** 10.3390/ijerph16183310

**Published:** 2019-09-09

**Authors:** Todd B. Ziegler, Chris M. Coombe, Zachary E. Rowe, Sarah J. Clark, Carina J. Gronlund, Michelle Lee, Angelina Palacios, Larissa S. Larsen, Tony G. Reames, Justin Schott, Guy O. Williams, Marie S. O’Neill

**Affiliations:** 1 School of Public Health, University of Michigan, Ann Arbor, MI 48109, USA (T.B.Z.) (C.M.C.); 2 Friends of Parkside, Detroit, MI 48213, USA; 3 Southwest Detroit Environmental Vision, Detroit, MI 48209, USA (S.J.C.) (A.P.); 4 Institute for Social Research, University of Michigan, Ann Arbor, MI 48109, USA; 5 Jefferson East Inc., Detroit, MI 48207, USA; 6 College of Literature, Science, and the Arts, University of Michigan, Ann Arbor, MI 48109, USA; 7 Taubman College of Architecture and Planning, University of Michigan, Ann Arbor, MI 48109, USA; 8 School for Environment & Sustainability, University of Michigan, Ann Arbor, MI 48109, USA; 9 EcoWorks, Detroit, MI 48219, USA; 10 Detroiters Working for Environmental Justice, Detroit, MI 48201, USA

**Keywords:** community-based participatory research, extreme heat, climate change, health equity

## Abstract

Extreme summertime heat is a significant public health threat that disproportionately impacts vulnerable urban populations. Research on health impacts of climate change (including increasing intensity, duration, and frequency of hot weather) is sometimes designed and implemented without the involvement of the communities being studied, i.e., “community-placed” not “community-based.” We describe how the Heatwaves, Housing, and Health: Increasing Climate Resiliency in Detroit (HHH) partnership engaged relevant communities by integrating a community-based participatory research (CBPR) approach into an existing, academic-designed research project through a steering committee of community and academic partners. Using a case study approach, we analyze program documentation, partnership evaluation questionnaires, and HHH steering committee meeting notes. We describe the CBPR process by which we successfully collected research data in Detroit during summer 2016, engaged in collaborative analysis of data, and shared results with Detroit residents. Evaluations of the partnership over 2 years show community involvement in research; enhanced capacities; success in securing new grant funding; and ways that CBPR strengthened the validity, relevance, and translation of research. Engaging communities as equal partners using CBPR, even after a study is underway, can strengthen research to understand and address the impacts of extreme heat on health and equity in urban communities.

## 1. Introduction

Due to climate change, extreme summertime heat is a significant and growing threat to public health. In US cities, disparities in health impacts related to extreme heat exist among racial, ethnic, and socioeconomic groups [1,2,3,4]. These disparities are often exacerbated by environmental conditions, with low-income, urban communities of color disproportionately impacted by neighborhood characteristics that contribute to urban heat islands and higher temperatures that can threaten health [5,6,7].

Scientists are developing multi-disciplinary approaches to model the health impacts of extreme heat in urban environments. In 2015, a research team from the University of Michigan (U-M), Georgia Institute of Technology (GT), and Arizona State University (ASU) was awarded a National Science Foundation (NSF) grant to estimate the health impacts associated with simulated energy grid failure events during heatwave conditions. The “3HEAT” study is a three-city study of Detroit, Phoenix, and Atlanta. The study aims to advance methodological innovation in the development of heat exposure metrics; quantify mortality and morbidity based on individually-experienced temperatures; link regional climate and building energy models, residential building stock, and grid infrastructure data; and compile data on current and future climate conditions. Additionally, the project will assess the effectiveness of specific environmental, technological, and behavioral adaptations in mitigating a growing heat hazard [8]. To satisfy this diverse set of research questions, the study had four tasks that required the involvement of local residents. These tasks were to (1) survey 100 Detroit residents to ask about their housing characteristics, heat-related behaviors, and demographics; (2) install temperature and humidity sensors inside and outside 50 Detroit homes; (3) ask the 50 residents living in the monitored homes to wear personal temperature monitors and record their activities and location for several ‘hot’ and ‘regular’ summer days; and (4) conduct semi-structured interviews at the end of the study to step through a three day blackout/heat wave event and ask residents to anticipate their actions and concerns throughout. While these data collection techniques may not seem invasive, they required high levels of trust between the study participants and research team. No more than minimal foreseeable risks to participants of the study and the community existed, and the relevant Institutional Review Boards (IRBs) approved the study.

NSF and other federal funders of environmental health research have increased efforts to directly involve community representatives in addressing complex environmental hazards. For example, the National Institute of Environmental Health Sciences (NIEHS) supports community–academic partnerships that seek to advance the understanding of environmentally-related diseases [9]. While many approaches to community-engaged research exist, each with widely varying levels of community ownership of and engagement in research, they are common in their shared view that impacted communities hold unique and critical forms of expertise which are essential to understanding and addressing the issue being studied [10]. Our case study focuses on community-based participatory research (CBPR), a partnership approach to research that equitably involves community members, organizational representatives, researchers, and others in all aspects of the research process, contributing expertise and sharing decision making and ownership of data between community members and academic investigators [11,12].

A study by O’Fallon & Dearry [13] highlights how specific NIEHS-supported research studies satisfy key principles of CBPR, including active collaboration and participation at every stage of research, fostering co-learning among both academic and community partners, ensuring projects are community-driven, disseminating results in useful terms, ensuring that research and intervention strategies are culturally appropriate, and defining community as a unit of identity. O’Fallon & Dearry conclude that CBPR “can be an effective tool to enhance our knowledge of the causes and mechanisms of disorders having an environmental etiology, reduce adverse health outcomes through innovative intervention strategies and policy change, and address the environmental health concerns of community residents.” [13] (p. 155) 

Recent studies also demonstrate how CBPR can strengthen scientific rigor, integrity, and the scientific enterprise itself [14,15]. Specific examples of CBPR and community-based approaches in climate change adaptation have highlighted the benefits and obstacles of enhancing capacity for these approaches; see, for example, Ebi & Semenza [16] and McClymont Peace & Myers [17]. Case studies also provide evidence that CBPR can be an effective approach in analyzing and addressing a broad range of environmental health disparities [18,19,20,21]. 

Challenges exist across the range of community-engaged research approaches, including CBPR. Critical issues for developing and following CBPR principles include: recognizing and addressing historical differences in power; establishing processes that ensure equity, shared influence, and control in decision making; balancing involvement of all partners with burden on partner time and resources; distributing resources equitably; and addressing conflicts associated with differences in perspectives, priorities, values, and language [11] (pp. 183–185) [12] (p. 278). Additional challenges specific to research design and methods include the need to identify and train community members in research processes, ensure credibility of the data, develop and disseminate materials to community and policy audiences in a timely and relevant manner, and balance time for data analysis with more immediate project needs [11]. Academic institutional barriers include the extensive time needed to conduct and publish rigorous research, limited funding to support community organizations’ participation, and the unfamiliarity of many IRBs with CBPR [10,22,23].

Factors that facilitate successful CBPR include the early involvement of community partners at the conceptual stages of a project, agreed upon guiding principles and operating norms to enhance equity, processes to overcome stereotypes held by community partners and academic researchers, acknowledgement of differences in how community-based organizations and academic institutions view research, and agreed upon objectives that address the priorities of both community and academic partners [24].

Many studies highlight the facilitating factors that contribute to successful CBPR implementation; however, few have examined how non-CBPR research projects can adopt CBPR approaches to strengthen research implementation, analysis, translation, and outcomes. Building off more than two decades of CBPR research and practice [25,26], which ideally involves starting a CBPR approach before grant funding is secured, we sought to address the following scientific question: “What is the process and what are lessons learned in a recent effort to integrate a CBPR approach into an existing non-CBPR study?”

Using a case study approach, we describe how the HHH partnership integrated a CBPR approach into the NSF-funded research based in Detroit, Michigan, including partnership formation, implementation, materials developed, and results of the CBPR research process. Next, we present methods and results from the evaluation conducted of the partnership process and outcomes. We assess the extent to which the objectives of the HHH are being accomplished and discuss the overall strengths and limitations of this study, the factors that contributed to our successes and challenges, and suggest recommendations for incorporating a CBPR approach into an established research study.

## 2. Formation of the HHH Partnership

The U-M research team involved in the 3HEAT project studies health issues related to climate change in Detroit using approaches from environmental health sciences, epidemiology, urban planning, meteorology, and social science, among other disciplines. Prior studies by U-M investigators highlight how environmental health issues are an environmental justice issue by revealing inequitable exposures and vulnerabilities to extreme heat conditions across city neighborhoods [7,27,28,29,30,31,32]. Community-based organizations (CBOs) in Detroit have extensive experience partnering with academics to conduct CBPR to address social and physical environmental health issues [33], and have also initiated diverse partnerships with academic researchers. 

In discussing implementation of the 3HEAT study, future community and academic members of the HHH partnership (including ZER, LSL, and others) recognized that community involvement would not only support the successful implementation of field work and data collection, but also enrich the analysis, interpretation and dissemination of data, and link research efforts directly with existing initiatives and policies designed to address climate adaptation and health equity issues. 

In accordance with the CBPR principle of building on strengths and resources within the community, the 3HEAT investigative team at U-M engaged representatives from local CBOs through conversations with the Detroit Community-Academic Urban Research Center (Detroit URC) [34]. The Detroit URC is a longstanding CBPR partnership among multiple Detroit organizations and academic researchers from U-M focused on health equity in the city of Detroit. The Detroit URC has extensive experience developing and implementing CBPR principles and protocols to build, foster and sustain long-term collaborative community-academic research partnerships and has fostered more than 75 CBPR partnerships and conducted over 100 research projects on critical health issues [33]. Drawing on this extensive experience, the U-M investigator of the 3HEAT study (MSO) consulted the Detroit URC Board about potential approaches, processes, and structures to equitably engage community organizations in the 3HEAT study. The Detroit URC board recommended the formation of the HHH steering committee with representatives from two Detroit URC board member organizations—Friends of Parkside and Detroiters Working for Environmental Justice—and three additional CBOs, Jefferson East Inc., Southwest Detroit Environmental Vision, and EcoWorks. Three of these CBOs are neighborhood specific (Friends of Parkside, Jefferson East Inc., and Southwest Detroit Environmental Vision), while the other two CBOs (Detroiters Working for Environmental Justice and EcoWorks) are umbrella organizations that focus on advancing environmental justice and sustainable and equitable energy provision throughout Detroit. 

The role of the steering committee was to guide implementation of the 3HEAT research project, integrate a CBPR approach into the existing study, ensure that the project addressed community-identified health equity issues related to extreme heat exposure, and foster long-term partnership development. The three neighborhood-specific organizations were engaged in recruiting, retaining, and disseminating information to the study participants, while the two umbrella organizations helped identify opportunities to shape local policy and disseminate information to local decision makers and stakeholders. Further, HHH established a co-PI structure, with a community member with over 20 years of experience as the community-PI (ZER) and the U-M researcher as academic-PI (MSO) (See Appendix A Table A1 for a full list of steering committee members). 

To support the implementation of a CBPR approach, the HHH partnership received funding from several sources, including the Detroit URC [34], Michigan Institute for Clinical and Health Research (MICHR) [35], the Michigan Lifestage Environmental Exposures and Disease (M-LEEaD) Center [36], and the Ginsberg Center for Community Service and Learning [37]. Our funders support multidisciplinary collaborations designed to address gaps in knowledge about lifestage vulnerability to environmental exposures; conduct translational science on topics of relevance to the communities involved; enhance the capacity of social sector community partners; and foster public engagement among U-M faculty, staff, and students. In all grants, CBOs had budget line items to pay for their time and effort as significant, ongoing research partners.

## 3. Carrying Out the 3HEAT Research Using CBPR

The overall aims of the HHH partnership are to (1) establish and maintain a steering committee, (2) build and/or enhance the capacity of both academic and community partners to apply a CBPR approach to the research project, and (3) evaluate the process, effectiveness, and outcomes of integrating a CBPR approach into an existing research project. We define capacity as the increased ability of both community and academic partners to engage in CBPR to understand and address community concerns. Additional goals of the HHH partnership include establishing short- and long-term goals of the research, creating field work protocols, identifying study participants, collecting and analyzing data, disseminating research results, defining partner roles, and developing additional processes for collaboration, including ground rules and decision-making protocols.

From 2016 to 2018, the steering committee met 26 times and community partners were represented at every steering committee meeting. To support equitable collaboration, the community and academic PIs, who are highly experienced in CBPR, facilitated the development of partnership protocols and operating norms, which included a consensus-based decision-making process, clearly defined roles of CBOs and academic partners, building the capacity of community and academic partners, and instituting communication and dissemination of results as standing meeting agenda items. We evaluated the extent to which there is shared power in communication and the steering committee collaboratively drafted working documents to support partnership development (see Appendix A Table A2 for additional details). 

### 3.1. Participant Recruitment & Field Work

The 3HEAT project aims to estimate temperature exposures in a more refined way than has been done previously, and to better understand the behaviors and perspectives of city residents regarding heat, health, energy infrastructure, and housing quality. Each of the three city sites proposed to survey city residents, conduct interviews, collect daily activity diaries, and collect temperature data from a variety of housing types. The proposed 3HEAT data collection approach prioritized a random sample of individuals stratified by residential building type and access to mechanical air conditioning (AC). After discussion, the steering committee concluded that a truly random sampling approach would have several disadvantages and likely not yield high participation rates. Rather, directly involving CBOs in the process and tapping into existing community networks would be a more effective form of recruitment. CBO partners shared the addresses of 100–125 potential participants who had been involved in prior programming or volunteer opportunities with the CBOs. The characteristics of the homes of these individuals were then classified by the U-M academic team using online tools, and a ranked list for recruitment was created with the goal of ensuring an equal distribution of housing types and AC access, in accordance with the original study design.

The U-M academic team, with guidance from the steering committee, developed a fieldwork manual containing detailed data collection protocols and “quick guides” to facilitate efficient and effective use of data collection monitors. Three Community Environmental Specialists (CESs) were hired and employed, one at each of the three CBOs representing the neighborhoods within the study (Southwest Detroit, Villages at Parkside, and Jefferson-Chalmers). CBO partners developed hiring protocols for CESs that were aligned with community norms and expectations. The U-M IRB approved the research protocol and all instruments for data collection from study participants (Approval # HUM00102979) and served as the IRB of record for all CESs as well as U-M personnel. All personnel received human subjects ethics training prior to commencing work involving human subject interactions. 

CESs and U-M field staff worked collaboratively but each had unique roles; CESs were generally responsible for direct interaction with program participants (e.g., recruitment, scheduling visits, consent of participants, conducting surveys) while U-M field workers were responsible for more “behind-the-scenes” data collection tasks (e.g., setting up temperature monitors, downloading data, providing transportation). CBO partners felt that hiring CESs directly through their organization supported the capacity of CBOs to train and hire community residents to develop new skills in research. One CES had a dual role and challenged the traditional community–academic dichotomy as both a CES and a U-M student (AP). AP had the unique ability to “bridge-span” [38] and as the child of new immigrants and a life-long resident of the Southwest Detroit neighborhood, AP was familiar with the challenges that many community members faced. After her first year in the university, AP worked for Southwest Detroit Environmental Vision for the summer helping the organization to address issues of air pollution, removing abandoned and blighted buildings, and creating community gardens and parks on vacant land. She joined the research project during summer 2016 to help collect, code, and interpret the data. AP’s lived experiences engendered trust from SW Detroit residents and helped balance data interpretation with local knowledge. 

CBO partners also supported the modification of surveys, time activity diaries and other data-collection tools by drawing from the local knowledge and expertise of community partners. In the summer of 2016, CESs recruited 53 Detroit residents who participated over a period of 8–12 weeks in the 3HEAT study. Of these participants, 29 participated in an in-depth interview, and in the spring of 2017, an additional 50 residents participated in the “survey-only” phase of the research.

### 3.2. Collaborative Analysis & Interpretation

The HHH steering committee collectively designed and conducted four internal workshops to adhere to key principles of CBPR, co-learning and building capacity among both community and academic partners. Academic partners presented a summary of the peer-reviewed literature on the health impacts of heat in Detroit. Community partners identified new research questions of relevance to community organizations, policy and other action opportunities that our research could support, and new audiences for research findings. Additionally, the workshops introduced community partners to basic epidemiological concepts, including the collaborative construction of a Directed Acyclic Graph which was designed to integrate local knowledge from community partners to identify new research questions on the links between reported heat-related health outcomes and housing features and access to or use of energy efficiency and weatherization services [39]. The steering committee also constructed a list of action opportunities to support the alignment of future dissemination materials with initiatives, policies, and other opportunities of relevance to community partners and residents.

### 3.3. Feedback of Findings to Participants

Using 3HEAT participant data collected in 2016, Individual Climate Reports were collaboratively developed and sent to study participants in the summer of 2017. These reports provided individualized study findings directly to program participants. The report included a comparison of residents’ personal, inside, and outside home temperatures with the average temperatures measured for all study participants, the U.S. Centers for Disease Control and Prevention recommended the summer indoor temperature range [40] and the temperatures measured at Detroit City Airport that summer. Academic and community partner steering committee members provided significant input on the content and design of the report. Additional resources were shared along with the report including a map and list of all known city cooling centers and factsheets providing general information on extreme heat and its impacts, best practices in identifying symptoms of heat-related illness, and strategies for coping with extreme heat. The goal of the report was to communicate individual- and community-level study findings back to participants, consistent with the CBPR principle of community access to and benefit from research results, and to encourage the continued engagement of participants in the interpretation and application of research findings.

HHH steering committee members also collectively developed and conducted three community-based focus groups in the summer of 2018. The goal of the focus groups was to report new research findings back to study participants, elicit suggestions to improve data reporting, identify recommended actions related to extreme heat and energy efficiency, and develop new tools to support community residents. Thirty-one participants from the original 3HEAT study participated. Initial research findings were shared directly with study participants, encouraging bi-directional dialogue between community residents and academic partners. After the focus groups, an analysis of summarized responses from participants was sent to all original 3HEAT study participants along with a newsletter highlighting the newly developed HHH partnership website [41]. While we did not directly measure the impact of these focus groups on participant knowledge, our CBO partners felt that participants gained a greater consciousness and understanding of extreme heat, protective measures, and broader environmental health issues. 

## 4. Methods & Materials

### Evaluation of the HHH Partnership

The partnership evaluation assessed its effectiveness in equitably engaging academics and communities in the research process for the 3HEAT study. Adhering to CBPR principles, we conducted participatory and developmental evaluation to assess partnership processes and outcomes towards achieving the HHH objectives stated above. The participatory approach, facilitated by an external evaluator (CC), involved the HHH steering committee and other participating faculty, students, and staff in a CBPR process to prioritize evaluation questions, identify indicators of success, and interpret and apply findings [42,43]. Partners’ active involvement in the evaluation enhances the relevance and increases the usefulness of the results [44]. As a formative evaluation, results were shared at steering committee meetings so that partners could interpret and apply the findings for learning and to build and strengthen the partnership [45].

The mixed methods evaluation [46,47] included ongoing program documentation (e.g., meeting minutes, participation logs, partnership protocols) and an annual partnership evaluation questionnaire completed by all steering committee members at the end of years 1 (2016–2017) and 2 (2017–2018). The questionnaire was based on a conceptual framework and existing surveys for evaluating CBPR partnerships [48,49] and adapted for the HHH partnership. Dimensions of partnership dynamics and effectiveness included participation, partnership goals and approach, organization, trust and mutual respect, decision making, resource sharing, communication, and benefits and challenges of participation. To modify the questionnaire for HHH, steering committee members discussed indicators of success and added closed-ended questions to assess community partners’ influence on the main aspects of the research process in conducting the 3HEAT study, and an open-ended question on ways in which using CBPR strengthened the research. Most closed-ended questions were rated on a 5-point Likert scale (e.g., from strongly agree to strongly disagree). Open-ended questions included partnership strengths, challenges, and lessons learned. 

The questionnaire was administered electronically using Qualtrics Survey Software, Version 2018 (Qualtrics, Provo, UT, USA). All 10 steering committee members (five academic and five community) completed the survey each year. We reported frequency and percent agreement (agree or strongly agree) for all items, compared years 1 and 2 for trends, and examined results by partner role (community or academic). Due to the small number of respondents (*n* = 10), we did not compute measures of central tendency, and responses were combined into three categories for reporting (strongly agree and agree, neither agree nor disagree, and disagree and strongly disagree). Qualitative data from open-ended questions were themed using a grounded theory approach to group content conceptually using verbatim language [50]. We examined findings from both quantitative and qualitative data and selected quotes to illustrate findings. Results were shared, discussed, and interpreted at steering committee meetings.

## 5. Results

The results are divided into five sections: (1) Partnership Dynamics and Effectiveness, (2) Community Influence on the Research Process, (3) How CBPR Strengthened the Research, (4) Strengths of the HHH Steering Committee, (5) Enhancing Capacity, and (6) Challenges. 

*Partnership Dynamics and Effectiveness*. Table 1 presents steering committee members’ assessment of key dimensions of the HHH partnership’s organization, processes, and functioning that contribute to effective and equitable CBPR partnerships [48].

Regarding partnership goals and approach, strong agreement was present in both years that HHH has the right partners around the table, holds shared goals, and is following CBPR principles and operating norms that enable the group to work together collaboratively. On core dimensions of equity, by year 2, 90% or more of steering committee members indicated high levels of having a voice in agenda setting, trust and mutual respect, shared decision making, and fair distribution of resources. While the steering committee was effective in communicating within the steering committee and between HHH members and their organizations (80–100% agreement), the HHH was less effective in communicating its work to the community and more broadly. This improved somewhat during the 2nd year, as the partnership became more established. (See Table 1 for full results).

*Community Influence on the Research Process*. Survey findings presented in Table 2 show steering committee members’ assessment of the extent to which community partners influenced different aspects of the research process, recognizing that the initial research study was designed without community input. 

Steering committee members indicated that community partners were substantially involved in developing plans for participant recruitment and data collection in both years. For most other activities, results show a trend toward greater involvement by the end of the 2nd year, as the research process progressed. The percent of steering committee members who indicated that community partners influenced the research process a great deal or much was highest for developing the recruitment plan (≥90%), informing or modifying process and protocols (67% year 1, 70% year 2), and collecting data (≥80%). Regarding decisions about resources, a key dimension of equity, by the end of the 2nd year, 80% of steering committee members expressed that community partners had influenced decisions about resources and budgets, compared to 44% in the 1st year. Only community involvement in applying research findings had low ratings in both years, a period during which the focus was on data collection and analysis.

*How CBPR Strengthened the Research. *Table 3 presents key findings from an open-ended question asking steering committee members to provide specific examples of ways in which the CBPR process strengthened the research. The results are presented as major themes that emerged from the participant responses, followed by specific responses that exemplify each theme of how using CBPR contributed to the research.

Overall, members indicated that engaging the community as partners both strengthened the validity of the research and made the study more relevant to the community. Community partners’ feedback improved data collection instruments, methods, and protocols that were initially researcher-developed. Steering committee members felt that all phases of data collection, including recruitment, participation, and retention of participants, were effective because of the community partners, including steering committee members, their organizations, and the CESs. 

As one academic steering committee member said, “Recruitment and retention was MUCH more efficient and effective with partners. I’m not sure we could have accomplished this at all [in Detroit] without them.”

*Strengths of the HHH Steering Committee*. In response to an open-ended question, participants described strengths of the steering committee, many of which are characteristics important for collaboration and participation in diverse groups [52]. Several themes emerged from participant responses, listed here. HHH brings together a group of community and academic partners that are diverse on multiple dimensions. Both academic and community partners, as well as staff, bring a range of talent, knowledge, and expertise to the partnership, as well as involvement in related networks and initiatives. A sense of trust, mutual respect, and shared investment exists. Partners expressed goodwill toward each other and the ability to work collaboratively in part from extensive CBPR experience among several members of the group. One partner stated, “HHH benefits from the high level of experience, prior relationships, and preexisting trust among many members.” The partnership recognizes the important challenge of balancing research, grant writing, and dissemination priorities. 

A further strength is the potential for HHH research to have an impact on a pressing issue that is gaining increased urgency—climate and health—as “various organizations … work collaboratively on data and solutions that will benefit future work and generations.”

*Enhancing Capacity.* Aim 2 of HHH is to build capacity and facilitate co-learning of both community and academic partners. The hiring of CESs directly through CBOs provided opportunities for community organization staff to learn new skills in research and strengthened the future research capacity for these organizations. The HHH steering committee also developed a data-sharing agreement with CBO partners, increasing CBO access to data to support community-driven research, programs, and policy advocacy efforts (see Appendix A Table A2). HHH steering committee members also met with the director of the City of Detroit’s new Office of Sustainability to identify potential new research questions or products that can support citywide sustainability efforts, including the Detroit Sustainability Action Agenda and other efforts to address priority issues identified by city officials, CBOs, and academic researchers. The partnership provided opportunities for one postdoctoral, three doctoral, eight masters level, and six undergraduate level students to engage in CBPR in both academic and community settings. 

In the 2nd year evaluation questionnaire, 50% or more of participants reported that participation in the partnership benefited them to a great or very great extent in the following ways: enhanced their ability to conduct CBPR, increased their professional skills and knowledge to address health-related issues, developed new collaborative relationships, increased resources for their organization, and provided new data they can use to understand and address community problems. Forty percent reported that participation increased their ability to inform policy change. (Data from these evaluation questionnaire items not presented in tables).

*Challenges*. Lack of sufficient and sustained funding was cited as a major challenge, contributing to disproportionate time spent on grant writing. Another challenge was the desire expressed by community partners to provide more direct benefit to the community, including education and intervention tools to protect vulnerable residents and work toward policy solutions. A particular concern was the delay by the academic team to get results and feedback back to community participants in a timely way. Some delays are inevitable because of data processing and competing demands, but the partnership agreed that more timely feedback is an area for improvement. Because the same people are very involved in many other activities in this arena, better alignment of HHH with existing initiatives or increased staffing is also needed.

## 6. Discussion

We have described the integration of a CBPR approach into a research study through the establishment of a partnership led by a steering committee consisting of community and academic representatives with a wide range of knowledge, expertise, and affiliations (see Appendix A Table A1). We report on ways in which community entities were engaged in the research process, including data collection, analysis, interpretation, and feedback of results to participants, and dissemination planning more broadly. We expected that a CBPR approach to data collection, analysis, interpretation, and dissemination would yield several benefits, including better success in recruiting and retaining research participants, higher quality data, and a more efficient translation of research findings to support the current and ongoing needs of CBOs and community residents themselves. We also anticipated better representation and integration of community needs and perspectives into the research process and future initiatives. We analyzed results of a partnership survey to assess the ways in which CBPR principles and practices affected partnership dynamics and effectiveness, influenced the research process, and enhanced the capacity of community and academic partners. Below we discuss the strengths and limitations of this study, the facilitating factors that contributed to our success, and the lessons learned and implications for successfully incorporating a CBPR approach into an established research study.

Our findings suggest that using a CBPR approach to more equitably involve communities most impacted by climate change in research strengthens the research process and increases its relevance to the community. Further, as we develop and strengthen CBPR protocols and equitable collaboration moving forward, we strive to embody core principles of environmental justice [51] by including representatives of impacted communities as equal partners in decision-making in research implementation, analysis, dissemination, and future actions. As one steering committee member noted, “I feel the CBPR approach has caused the project to be far more successful in generating awareness at the neighborhood and community level, and set the stage for leveraging the research findings into strong and relevant policy ideas.”

### 6.1. Strengths 

Use of CBPR has enabled and strengthened opportunities for community partners to engage in multiple aspects of the research process, including data collection and analysis, interpreting results, and translating and disseminating findings into useful tools. HHH steering committee members’ assessment of their influence in decisions about resources and budgets showed an upward trend, as did their belief that the appropriate partners are engaged in this research partnership and members hold shared goals, decision making, trust, and mutual respect. Although the small number of participants did not allow us to determine if changes are statistically significant, the positive trend is encouraging when combined with qualitative findings. We have also used our evaluation findings to identify and address needs for improvement, for example, to better communicate our successes to outside partners. 

A major strength of using CBPR was the increase in opportunities for community organizations to engage in climate adaptation research that can inform individual and policy change. Our CBO partners expressed that they are addressing a relevant issue in their community and that academic partners have shown a genuine willingness to work equitably toward shared power and control of the research agenda. HHH partners have also collaboratively developed proposals for longer-term research projects to address climate change. 

### 6.2. Challenges 

In addition to the strengths of using this approach, we experienced a number of challenges. Holding focus groups with prior study participants has provided opportunities for continued engagement, including direct feedback by residents on dissemination materials and identification of additional community-level environmental health concerns. A challenge of the CBPR process that may have impacted participation in our focus groups was the 1-year delay between data collection and feedback to participants. Community partners expressed dissatisfaction with the slow pace of the process, and the partnership has made some progress on making dissemination materials more accessible to residents sooner in the future. Another significant challenge was related to data collection tools, which were designed for the initial study by researchers without community input. For example, community partners found the Time Activity Diary tool to be complicated even after substantial revisions. Given a tight timeframe—the need to conduct the research during the summer using tools approved by all three university IRBs—this tool was deployed despite its flaws, and some study participants had difficulty accurately filling it out. However, other 3HEAT study groups in Atlanta and Phoenix, who did not engage in a CBPR process, encountered similar challenges. 

Additional challenges were related to the development of dissemination materials that were deigned to communicate the significance of individual study participants’ temperature results directly back to them. We found that results of a single research study may be insufficient to make clinical or policy recommendations without further research [53]. In the case of the Individual Climate Report, we provided indoor temperature ranges, but were not able to provide concrete recommendations on “safe” indoor temperature ranges outside of those available for occupational settings or within the context of particular existing health problems.

Priorities and timeframes often differ significantly between our academic and community partners. Academic partners often have limited time to work on policy or program development if a research component is absent. Community partners may find it difficult to justify contributing to research if the applications and direct benefits to the communities they serve are not clear or timely. Using a formative evaluation approach has provided our partnership with opportunities to identify tensions and overlap between the goals of academic and community partners and to align our efforts to better meet mutual goals, even when these goals lie beyond the usual core areas of focus for each individual partner. 

Moving forward, our partnership is focused on translating our data and expertise to advance health and energy efficiency, affordability, and equity. In accordance with CBPR principles, the steering committee will continue to pursue opportunities to apply findings from this research to advance interventions and policies to improve Detroit communities. 

### 6.3. Recommendations 

For those seeking to incorporate CBPR approaches in other projects, based on our experiences, we recommend the following [26]: (1) Assure that community partners and study participants are adequately compensated for their time and expertise. Conversations on grant funding allocation should take place during partnership formation and maintenance; (2) Establish multiple pathways for feedback and communication, such as a 5–10 min debrief session following meetings focused on satisfaction with the conduct of the meeting, as well as annual surveys on partnership satisfaction; (3) Build on existing partnerships, when possible; (4) Develop a consistent strategy for dissemination that includes community-oriented, non-academic avenues; and (5) Equitably share the decision-making and the research agenda among community and academic partners.

Ideally, a CBPR approach would be used from the beginning of the research process and involve community partners in defining the research questions and designing the study methodology. In our case, we found that the value in integrating CBPR into the existing 3HEAT study was to improve data collection, analysis, interpretation, and dissemination. Additionally, using CBPR entails a commitment to engage communities equitably in decision making in subsequent proposal development. HHH has attempted this and has submitted several grant proposals, all of which included community partners from the start.

Further, adhering to the CBPR principles of applying research findings for action at multiple levels—including interventions, programs, and policies—and having a long-term commitment to the work, means that efforts will extend beyond a specific grant period. Thus, our partnership is committed to working towards balancing research and action. A challenge inherent in conducting research and then translating research findings into interventions and policies is related to the time needed to comply with scientific standards of rigor and reporting to get research results, and the need to be agile and responsive to policy and community needs which are often more immediate. This has been well-documented in the literature as an ongoing challenge in CBPR, particularly with newer partnerships that have not yet built the body of findings, processes, and skills to be more agile. HHH is committed to achieving this balance to ensure that both community and academic needs are being met. 

Another potential limitation relates to who is, and who is not, participating in our partnership. The CBO partners in HHH cannot represent all community perspectives or points of view, but their participation, and their connection to a broad range of networks working on related issues (see Appendix A Table A1), adds a value and depth to our research that would otherwise be missing. 

Ultimately, the HHH partnership highlights how research projects that are designed without substantial input of community-based partners can still bring community partners to the table in equitable and consequential ways. In the voice of one steering committee member, our partnership is a “demonstration that funders, community building organizations, and residents can work together to implement research that affects them directly.”

## 7. Conclusions

Addressing the impact of climate change in low-income, urban communities is an important health equity issue, and CBPR can be an effective approach to introduce into non-CBPR research focused on environmental health disparities. While ideally CBPR strives to engage communities equitably in all aspects of research from the beginning, our case study demonstrates the value of integrating aspects of CBPR to strengthen outcomes, identify mutually beneficial goals, build long-term equitable research partnerships, and promote the inclusion of community perspectives in basic science studies. Such an approach facilitates identifying and addressing immediate climate change-related environmental health needs of vulnerable communities; in this case, working toward assessing the need for and access to comfortable, healthy indoor environments (e.g., homes or public spaces with air conditioning, shading, or proper insulation to maximize thermal comfort). 

Shifting from “community-placed” to “community-based” participatory research provides opportunities to enhance the capacity of both community and academic partners, build trust between academic institutions and communities, integrate local knowledge to strengthen and enrich analysis, and interpret and disseminate research findings in ways that are more relevant and responsive to impacted communities. While developing CBPR processes to guide research takes additional time, engaging in CBPR has the potential to enrich research in ways that may not be possible without community input. Community partners not only know the needs of the community, how to engage the community, and the context for the data being collected, but also have expertise in policy and action-oriented opportunities at the local level. The experience of the HHH partnership described here may inform others who wish to adopt similar approaches with the goal of enhancing the quality and impact of research to address climate change.

## Figures and Tables

**Table 1:** HHH CBPR partnership dynamics and effectiveness: Results from the HHH Partnership Evaluation Questionnaire Years 1 and 2.

Partnership Dimensions and Questionnaire Items	2016–2017	2017–2018
	*N* = 10	*N* = 10
	Agree or Strongly Agree *n*	Agree or Strongly Agree *n*
Partnership Goals and Approach		
There is general agreement about the goals and objectives of HHH.	9	9
The steering committee has the appropriate members around the table in order to achieve our goals.	10	10
Steering committee members share responsibility for getting the work done.	10	9 *
Our operating norms enable partnership members to work together collaboratively.	9	9
Overall, the partnership is following its own community-based participatory research principles.	8	9
Overall, HHH is effective at accomplishing its goals and objectives.	9	8 *
Organization and Effectiveness of Meetings		
I find HHH steering committee meetings to be useful.	8	9
The steering committee meetings are well organized.	9	9
If I want to place something on a meeting agenda, I am comfortable with the process for doing so.	9	9 *
The opinions of some individuals are weighted more heavily than they should be.	1	1
I contribute actively to discussions during HHH steering committee meetings.	9	10
Trust and Mutual Respect		
I can talk openly and honestly at meetings.	10	10
HHH steering committee members respect each other’s points of view even if they might disagree.	10	10
There is a high level of trust between steering committee members.	9	10
The group values everyone’s knowledge and expertise.	9	9
Decision Making and Resource Sharing		
All steering committee members have a voice in decisions made by the group.	9	10
I am satisfied with the process by which the steering committee makes important decisions.	8	9
Steering committee members work well together to solve problems.	9	10
Decisions about resources are made in a fair manner.	8	9
HHH resources are shared in a fair manner.	8	10
I am satisfied with the amount of influence that each partner has over decisions that the steering committee makes.	8	9
Leadership between community and research partners is shared.	8	8
Communication		
Steering committee members communicate effectively with each other during meetings.	8	10
I communicate the priorities and needs of my constituency to the HHH steering committee.	8	9
I communicate about the work of HHH to my organization.	8	10
I communicate about the work of HHH to my community/constituency.	2	8
HHH communicates its successes effectively.	3	5

Note: Questionnaire instructions stated: Please indicate the extent to which you agree or disagree with the following statements (Strongly Agree, Agree, Neither Agree nor Disagree, Disagree, Strongly Disagree). * *n* = 9.

**Table 2:** Community influence on the research process: Findings from the HHH Partnership Evaluation Questionnaire years 1 and 2.

Questionnaire Instructions Stated: Please Indicate the Extent to Which Community Partners Influenced the Following Aspects of the Existing Research Process. (A Great Deal, Much, Some, A little, Not at all)	2016–2017 (*N* = 9)	2017–2018 (*N* = 10)
**Components of the Research Process**	**A Great Deal or Much** ***n***	**A Great Deal or Much** ***n***
Develop the plan to recruit, inform, and retain participants	9	9
Inform/modify data collection process and protocols	6	7
Collect data (implement field work)	8	8
Analyze data (compile and analyze data)	3	7
Interpret results (feedback and discuss)	2	6
Translate and disseminate findings (translate into useful tools and communicate with diverse audiences)	2	7
Apply findings to interventions and policies (*n* = 7)	2	3
Evaluate partnership process, effectiveness, and outcomes	5	8
Decide on resources/budget modifications	4	8

**Table 3:** How using CBPR strengthened the research: Emergent themes followed by examples of verbatim responses from partnership evaluation survey results, Years 1 and 2 combined.

Engaging community throughout has strengthened the validity of the research.
Coordination between staff and the input and feedback from community partners throughout has made the project stronger.
CBPR has strengthened the validity of the research by incorporating the communities’ inputs.
Community partners have made the study more relevant to the community.
Community partners bring relevance and humanity to the processes. Without connecting with residents and organizations, how do you make the study relevant to the community and populations the study will inform.
The CBPR approach has made the content relevant to our homeowners.
Recruitment, participation, and retention of residents was effective because of community partners.
Hiring community environmental specialists made recruitment, data collection, and field work more community-driven and sensitive to community needs.
Helped secure participation of multiple Detroit residents, get a good participation rate.
Researchers would have had a harder time establishing credibility in recruiting and enrolling participants.
Community partners’ feedback improved data collection instruments and methods.
Community partners’ feedback on the survey and time-activity-diary instruments was helpful.
Burdensome questions and data collection methods were avoided.
Translation and feedback of results to participants was more accessible.
Community partners helped translate language and study actions to be more accessible to residents.
Designing individual climate reports, focus groups & dissemination of results to participants.
Collaborative analysis exercise strengthened analysis and interpretation of data.
Co-analysis exercise focused the research question and analysis.
Collaborative analysis and interpretation of data, and future dissemination made research more accessible.
Findings can inform individual, community, and policy change.
Allowed residents to take a part in climate change activity that they potentially would not have had this opportunity.
Our study findings have a better opportunity to connect with existing policy initiatives and identify action opportunities.
Collaborative process resulted in grants, proposals, and concepts for sustainable partnerships.
Acquired pilot grants that will allow for co-analysis, co-interpretation, and dissemination that is more responsive to community needs and CBO capacity.
Collaboratively developed large-scale proposals and concepts for future sustainable partnership, even though not funded.
The potential for future partnerships and collaboration is strong.
Involvement of impacted communities in all aspects of the research is essential to environmental justice.
Adhering to the Principles of Environmental Justice ^1^, CBPR is essential for any research involving impacted communities; community members always need to have an impactful role in all processes.

^1^ The 17 Principles of Environmental Justice were developed and adopted by delegates to the First National People of Color Environmental Justice Summit in 1991. The principle referred to here is Principle 7 that states that “Environmental Justice demands the right to participate as equal partners at every level of decision-making, including needs assessment, planning, implementation, enforcement, and evaluation” [51].

## Appendix A

**Table A1:** HHH Steering Committee members.

Member Name	Affiliation	Expertise	Relevant Affiliations
Marie O’Neill	School of Public Health	Environmental epidemiology;	Detroit URC Board; M-LEEaD Center
(Academic PI)		environmental health	
Carina	Institute for Social	Environmental	Detroit City Council Green Task Force;
Gronlund	Research	epidemiology	M-LEEaD Center
Larissa Larsen	Taubman College of	Environmental planning;	U-M Poverty Solutions; Detroit Climate
	Architecture and	urban climate; water	Action Collaborative; Detroit Sustainability
	Urban Planning	infrastructure	Action Agenda
Tony Reames	School for	Energy equity; environmental	Energy Waste Reduction Low-income
	Environment &	justice	Workgroup; Detroit Climate Action
	Sustainability		Collaborative; Graham Institute; U-M Energy
			Institute; U-M Center for Local, State, and
			Urban Policy; The Ecology Center
Todd Ziegler	School of Public Health	Program coordination	The Ecology Center (Health Leaders
			Fellowship)
Zachary Rowe	Friends of Parkside	Community-based	Detroit URC Board; Healthy Environments
(Community PI)		participatory research;	Partnership
		public housing; social services	
Michelle Lee	Jefferson East Inc.	Housing, community	Community Development Advocates of
		engagement	Detroit; Wayne State University AmeriCorps
			Urban Safety Project; Community Economic
			Development Association of Michigan; City of
			Detroit Planning & Development; Housing
			Revitalization Department; Detroit Land Bank
			Authority; Detroit Office of Sustainability;
			Wayne State University Healthy Homes
			and Urban Safety; Rebuilding Together
			Southeast Michigan; Michigan State Housing
			Development Authority
Sarah Clark	Southwest Detroit	Urban planning;	Detroit Environmental Agenda; Southwest
	Environmental Vision	environmental health	Detroit Eco-D
Guy Williams	Detroiters Working for	Green jobs; environmental	Detroit URC Board; Future Build Construction
	Environmental Justice	policy	Group; Detroit Sustainability Action Agenda;
			Detroit Environmental Agenda; Detroit City
			Council Green Task Force; Center for Urban
			Responses to Environmental Stressors
Justin Schott	EcoWorks	Energy efficiency,	Michigan Energy Efficiency for All; Eco-D;
		environmental policy	NOAA Climate Literacy Grant; Detroit
			Sustainability Action Agenda; Detroit
			Environmental Agenda; Coalition to Keep
			Michigan Warm; 2030 District; Enterprise
			Community Partners; Detroit City Council
			Green Task Force
Chris Coombe	School of Public Health	Community-based	Detroit URC; M-LEEaD Center
(Evaluator)		participatory research;	
		partnership evaluation	

**Table A2:** HHH partnership development working documents.

Domain	Document	Description
Partnership	Partnership Ground	Exercise to identify group characteristics that contribute to successful
Development	Rules & Operating	Partnership.
	Norms	
	Decision Making	Group recognizes the 70% rule, a consensus-based decision-making process
		used by the Detroit URC. During the decision-making process, partners are
		to ask whether they can get 70% behind a decision; if participants are less than
		70% in support, the decision should be discussed more. If there is
		disagreement, and the issue is time-sensitive, there is an obligation to share
		what is blocking your decision in order to quickly resolve the issue.
	Scope of services:	Defines the roles and expectations of steering committee members, short- and
	Program Steering	long-term goals, and CBPR protocols and partnership strategies.
	Committee Membership	
Field Work &	Scope of Work:	Describes roles and responsibilities for CESs and U-M field staff and overview
Data	Community	of field work.
Collection	Environmental	
	Specialists & U-M Staff	
	Screening &	Describes timelines and expectations for study participant recruitment.
	Recruitment Protocols:	
	NSF Program	
	Implementation	
	Work Agreement:	Describes CBO responsibilities and target numbers for each CBO in the
	Community-Based	recruitment and implementation of data collection.
	Organizations (CBOs)	
	Participant	Describes the compensation schedule for study participants.
	Compensation Budget	
	Field Work Training	Training agenda for CESs and participating CBOs in all field data collection
	Agenda (CES)	processes and logistics.
	Program	Provides timeline for IRB approval, recruitment, enrollment data collection,
	Implementation	and participant follow-up.
	Timeline	
Analysis &	Data Sharing	Data use privacy pledge agreement for the sharing of data between U-M and
Interpretation	Agreement	CBO partners.
	Action Opportunity	Table of community-identified policy and other action opportunities that could
	Table	benefit from HHH research.
	Directed Acyclic Graph	Epidemiological model that integrated community-input to evaluate
		community-identified variables contributing to heat-related health outcomes.
Partnership	Qualtrics Survey	Survey questionnaire adapted by HHH from existing questionnaires to
Evaluation		evaluate the process, effectiveness, and outcomes of the partnership steering
		committee, and to inform and improve CBPR processes moving forward.
